# Supplementary material for: Accurate nonlinear mapping between MNI volumetric and FreeSurfer surface coordinate systems
Source: Hum Brain Mapp. 2018 May 16;39(9):3793–808. doi: 10.1002/hbm.24213 (PMC6239990; doi:10.1002/hbm.24213)
Supplement: Supplementary file 1 — Supporting Information [file HBM-39-3793-s001.pdf]

# SUPPLEMENTAL FIGURES

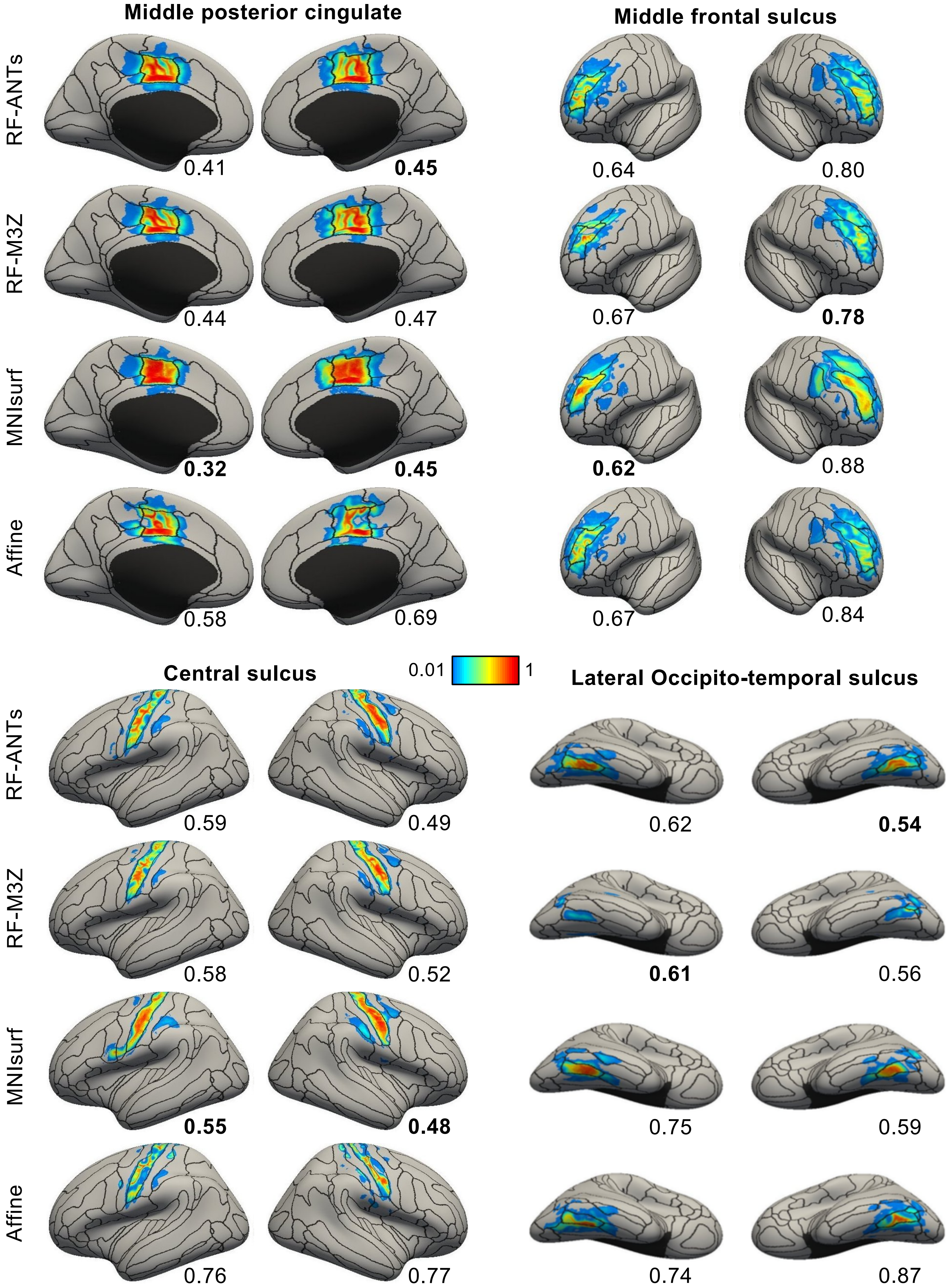


**Figure S1.** Visualization of ANTs-derived Colin27 probabilistic maps projected to fsaverage surface space in the GSP test set. Four representative structures are shown. Black boundaries correspond to the "ground truth" winner-takes-all parcellation. The value below each cortical surface shows the Normalized Absolute Difference (NAD) between projected probabilistic map and "ground truth" probabilistic map, where a smaller value indicates better performances. Best NAD for each region is **bolded**.

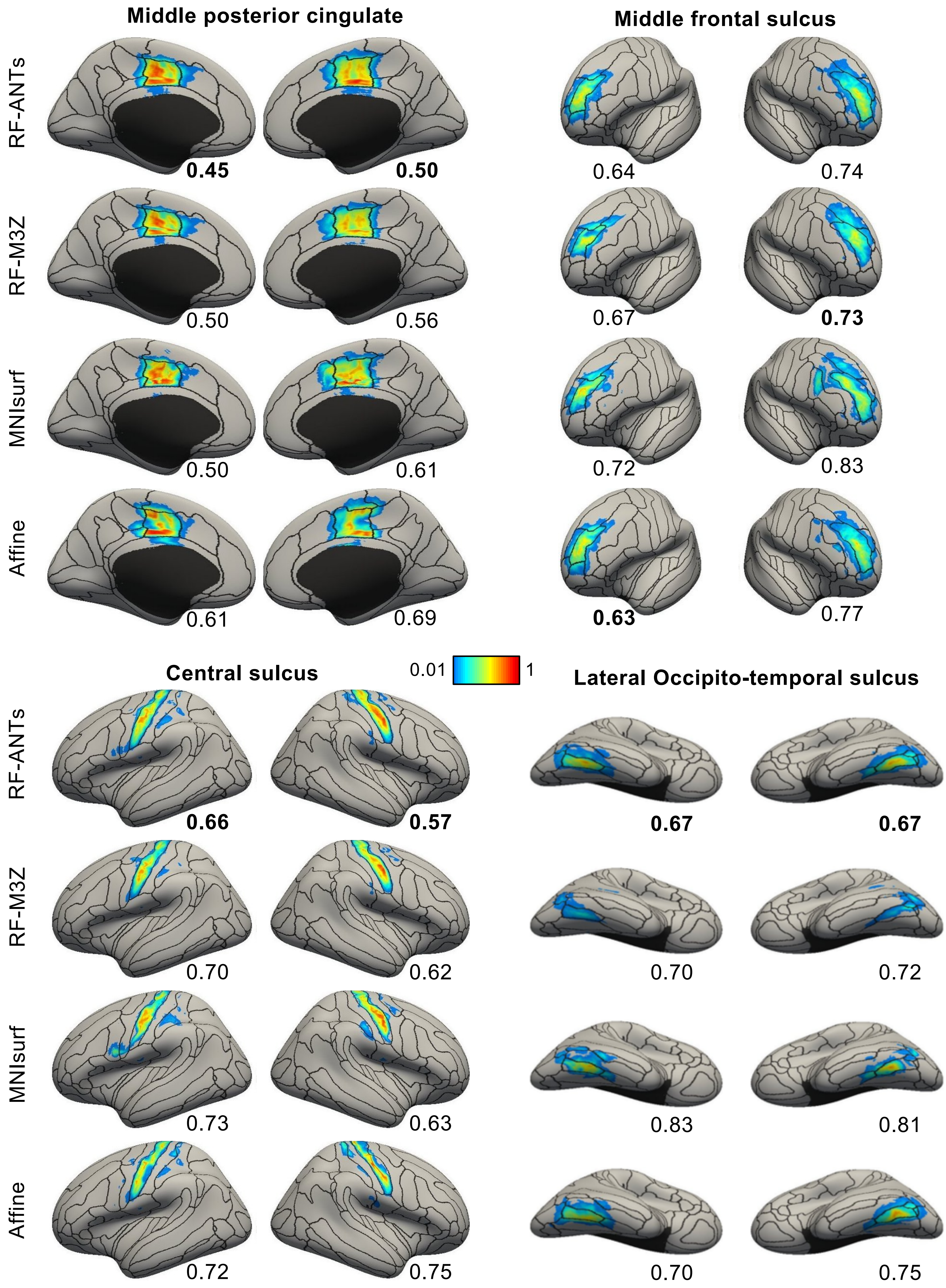


**Figure S2.** Visualization of FNIRT-derived Colin27 probabilistic maps projected to fsaverage surface space in the CoRR-HNU dataset. Four representative structures are shown. Black boundaries correspond to the "ground truth" winner-takes-all parcellation. The value below each cortical surface shows the Normalized Absolute Difference (NAD) between projected probabilistic map and "ground truth" probabilistic map, where a smaller value indicates better performances. Best NAD for each region is **bolded**.

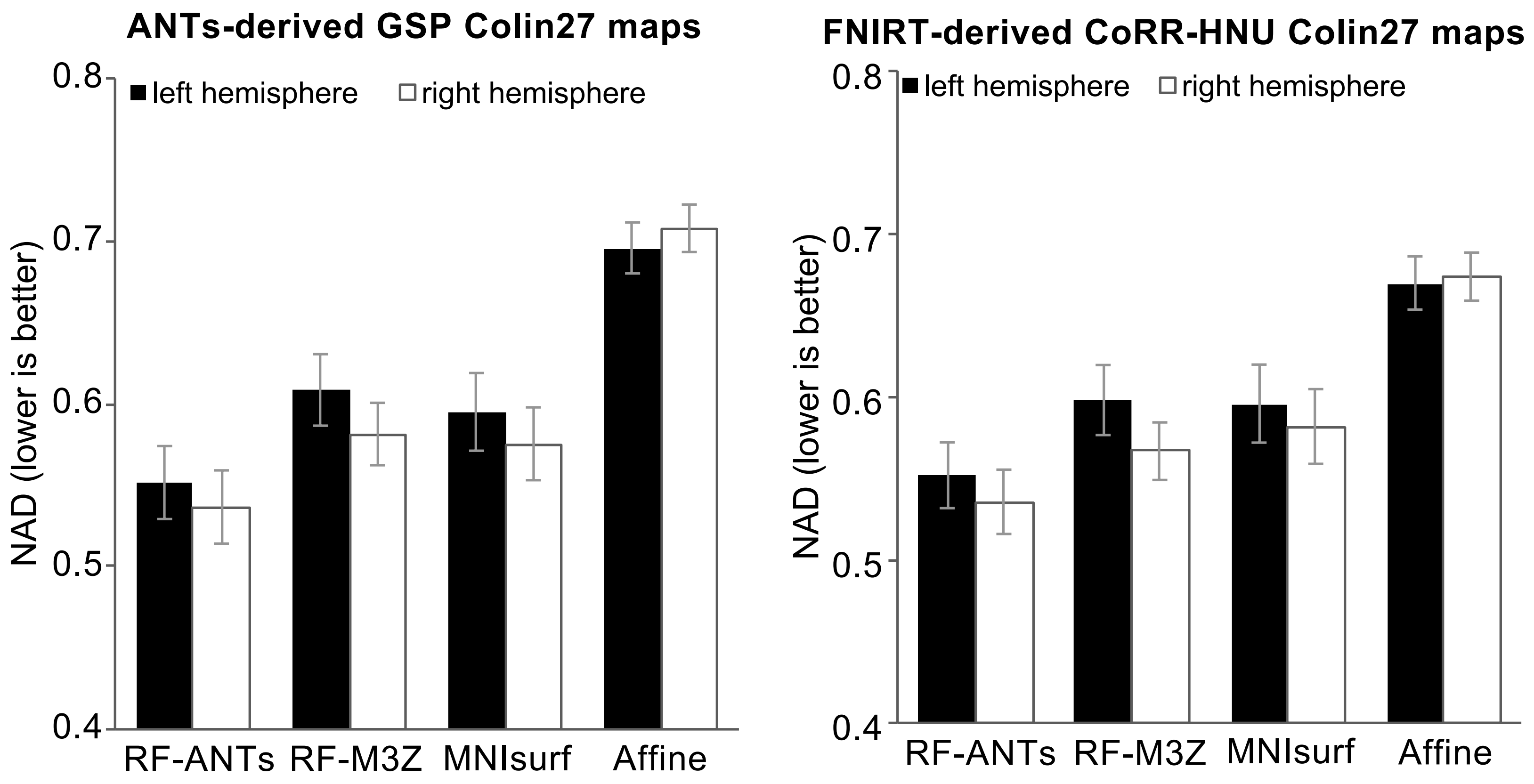


**Figure S3.** Normalized Absolute Difference (NAD) of Colin27 probabilistic maps projected to fsaverage surface space. (Left) Results for ANTs-derived GSP MNI152 probabilistic maps. (Right) Results for FNIRT-derived CoRR-HNU Colin27 probabilistic maps. The bars represent the NADs averaged across all 74 probabilistic maps within left hemisphere (black) and right hemisphere (white). Error bars correspond to standard errors across the 74 anatomical structures. Overall, RF-ANTs performed the best. Numerically, it may seem that the right hemisphere show smaller NAD values for the nonlinear methods  and larger NAD value for Affine, compared to the left hemisphere. However, the difference were not significant (all $p > 0.1$).

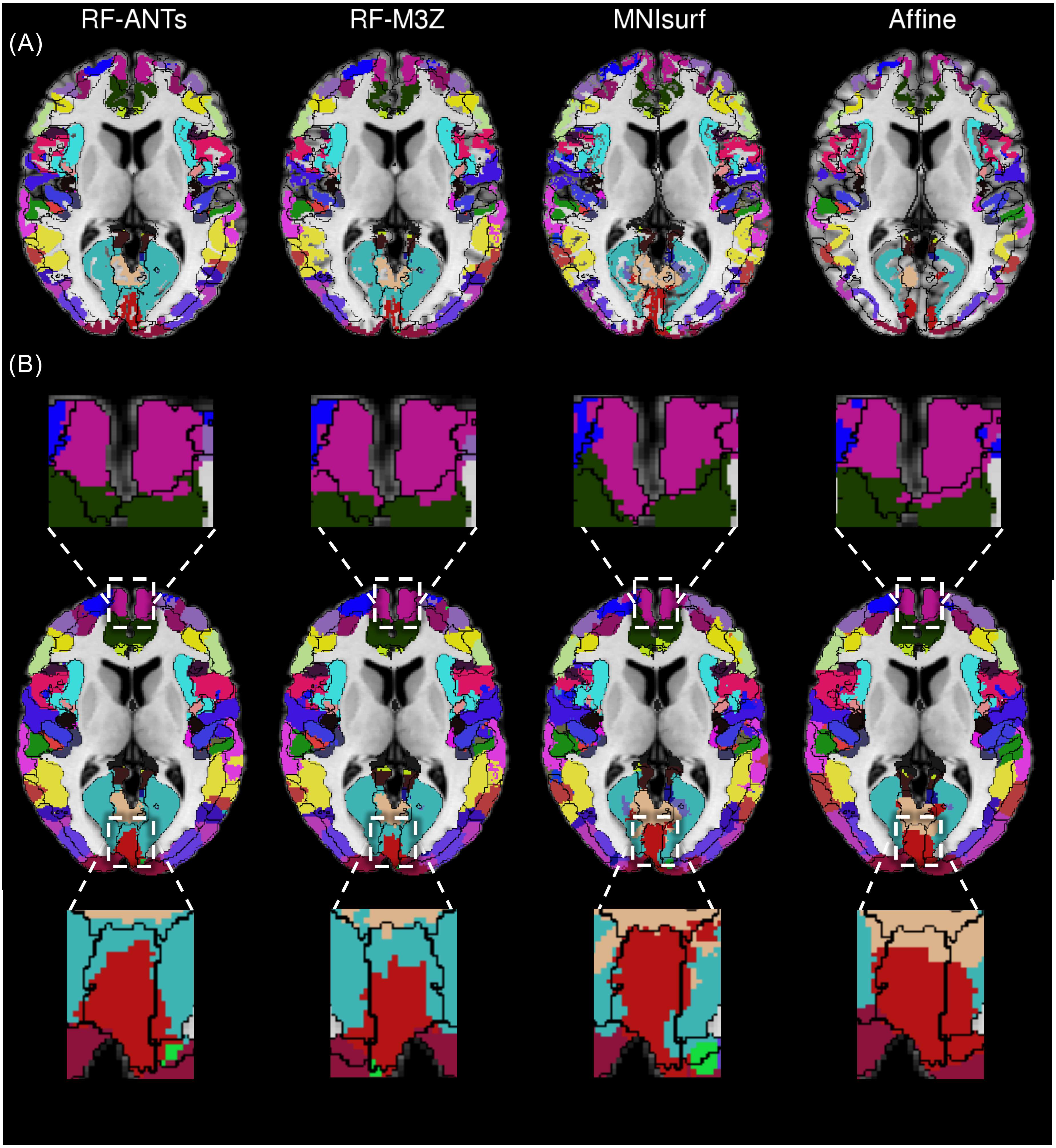


**Figure S4.** Winner-takes-all fsaverage parcellation projected to Colin27 volumetric space with ANTs-simulated "ground truth" (black boundaries) in the GSP test set. (A) Projections before dilation within loose cortical mask. (B) Projections after dilation within loose cortical mask.

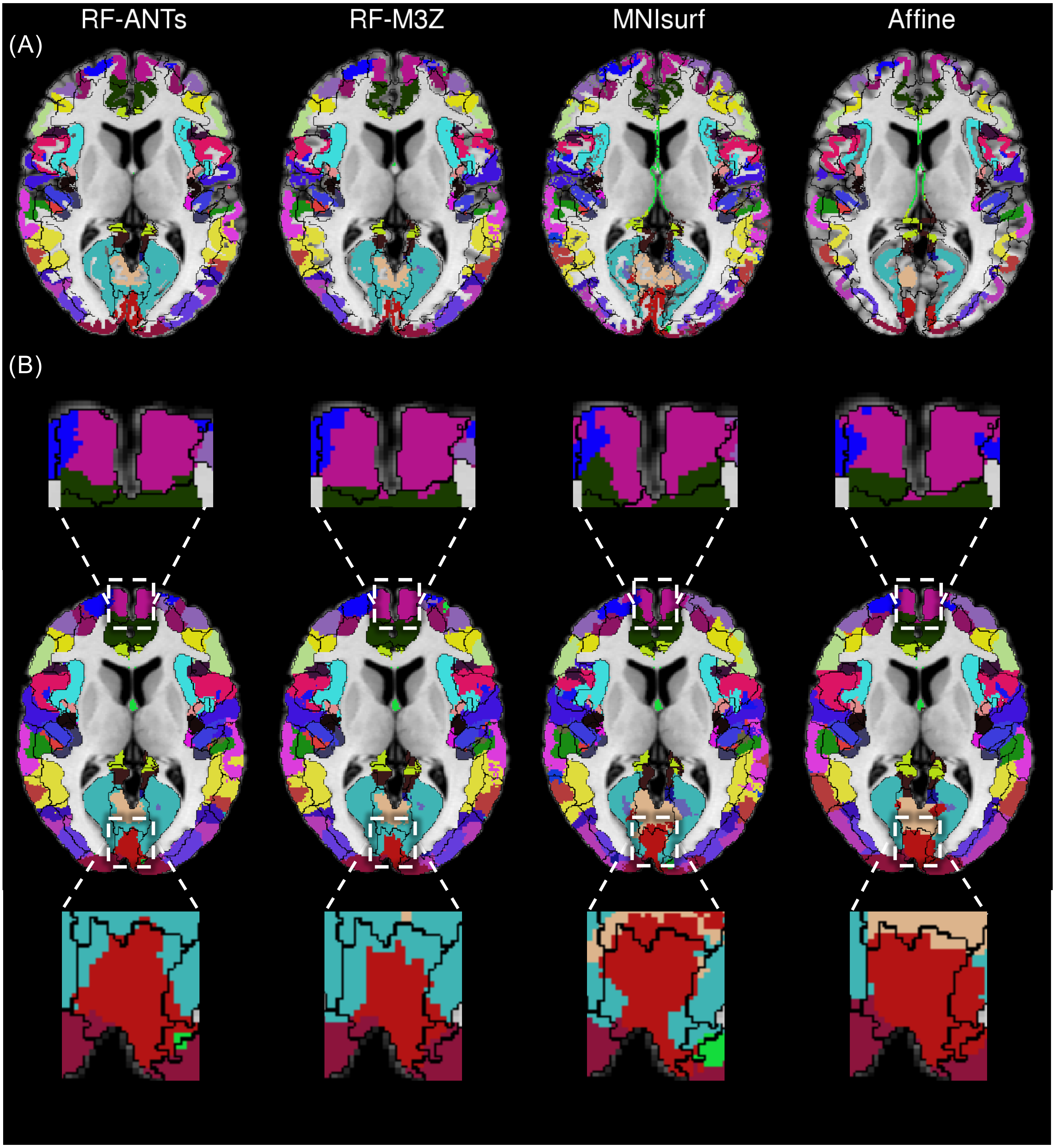


**Figure S5.** Winner-takes-all fsaverage parcellation projected to Colin27 volumetric space with FNIRT-simulated "ground truth" (black boundaries) in the CoRR-HNU dataset. (A) Projections before dilation within loose cortical mask. (B) Projections after dilation within loose cortical mask.

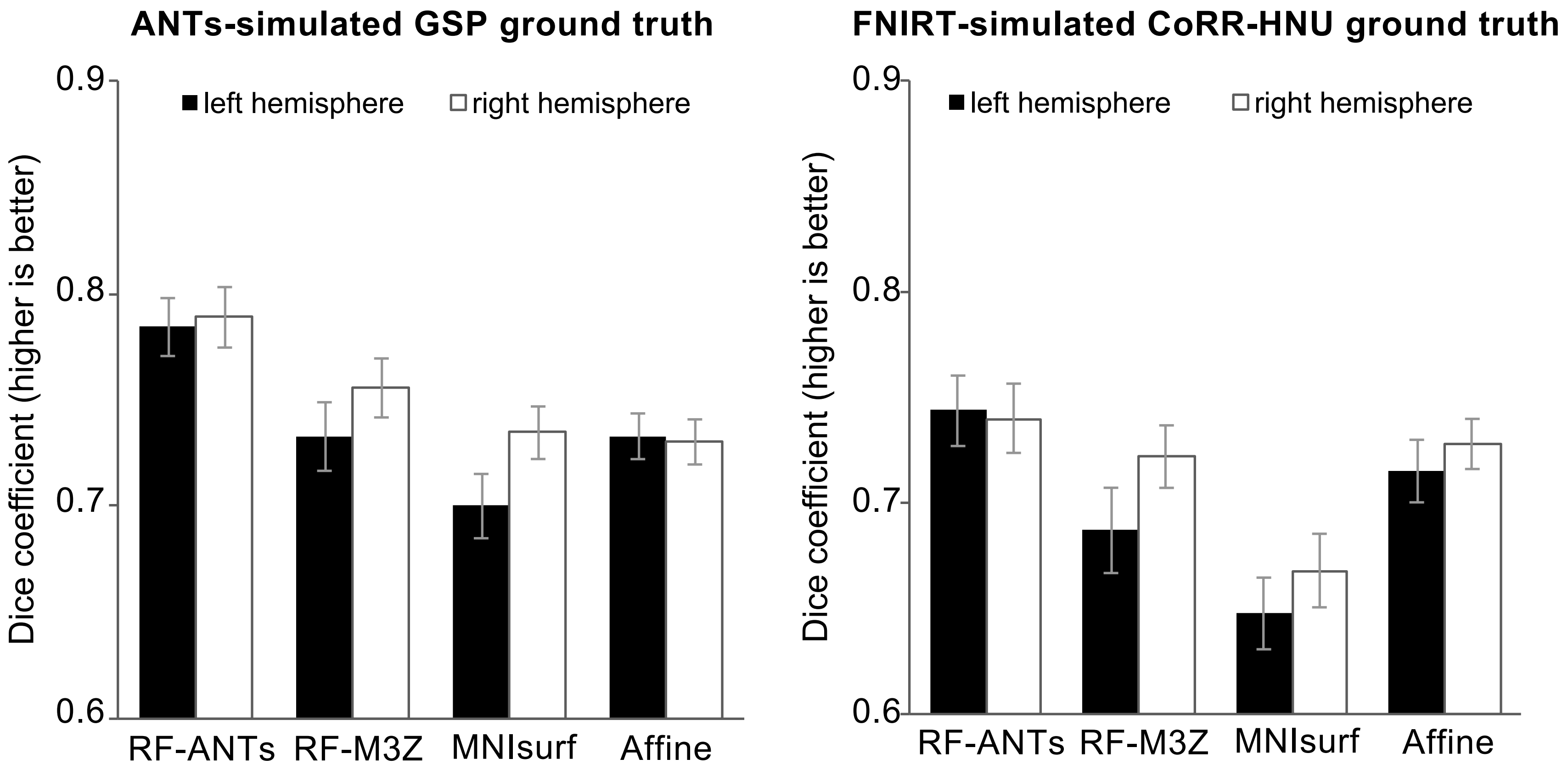


**Figure S6.** Dice of winner-takes-all fsaverage parcellation projected to Colin27 space, compared against (left) ANTs-simulated GSP "ground truth" and (right) FNIRT-simulated CoRR-HNU "ground truth". Bars represent Dice coefficient averaged across all 74 segmentation labels with left hemisphere (black) and right hemisphere (white). Error bars correspond to standard errors.
